# Recombinant Zika NS1 Protein Secreted from Vero Cells Is Efficient for Inducing Production of Immune Serum Directed against NS1 Dimer

**DOI:** 10.3390/ijms19010038

**Published:** 2017-12-23

**Authors:** Wildriss Viranaicken, Alexia Ndebo, Sandra Bos, Philippe Souque, Gilles Gadea, Chaker El-Kalamouni, Pascale Krejbich-Trotot, Pierre Charneau, Philippe Desprès, Marjolaine Roche

**Affiliations:** 1 Processus Infectieux en Milieu Insulaire Tropical (PIMIT), Université de La Réunion, INSERM UMR 1187, CNRS 9192, IRD 249, Platform CYROI, 2 rue Maxime Rivière, 97491 Sainte-Clotilde, La Réunion, France; wildriss.viranaicken@univ-reunion.fr (W.V.); Alexia.Ndebo@etu.unige.ch (A.N.); sandrabos.lab@gmail.com (S.B.); gilles.gadea@inserm.fr (G.G.); Chaker.El-Kalamouni@univ-reunion.fr (C.E.-K.); pascale.krejbich@univ-reunion.fr (P.K.-T.); 2 Section des Sciences Pharmaceutiques, Université de Genève, rue Michel-Servet 1, 1211 Genève, Switzerland; 3 Virologie Moléculaire et Vaccinologie, Institut Pasteur, 28 Rue du Dr Roux, 75724 Paris CEDEX 15, France; philippe.souque@pasteur.fr (P.S.); pierre.charneau@pasteur.fr (P.C.)

**Keywords:** emerging disease, arbovirus, Zika virus, NS1 protein, lentiviral vector, recombinant antigen, humoral immunity, NS1 antiserum

## Abstract

Zika virus (ZIKV) is a mosquito-borne flavivirus that recently emerged in the South Pacific, Americas, and Caribbean islands, where the larger epidemics were documented. ZIKV infection in humans is responsible for neurological disorders and microcephaly. Flavivirus NS1 is a non-structural glycoprotein that is expressed on the cell surface and secreted as a hexameric lipoprotein particle. Intracellular NS1 exists as a dimer that is required for viral replication, whereas the secreted NS1 hexamer interacts with host factors, leading to pathophysiological conditions. In an effort to dispose of specific anti-ZIKV NS1 immune serum, Vero cells were transduced with a lentiviral vector containing the NS1 gene from an epidemic strain of ZIKV. We showed that stably transduced Vero/ZIKV NS1 cell clone was efficient in the secretion of recombinant NS1 oligomer. Immunization of adult rat with purified extracellular NS1 developed anti-ZIKV antibodies that specifically react with the NS1 dimer produced in human cells infected with African and Asian strains of ZIKV. The rat antibody against ZIKV NS1 dimer is a reliable biological tool that enables the immunological detection of secreted NS1 from host-cells infected with ZIKV.

## 1. Introduction

Emerging mosquito-transmitted Zika virus (ZIKV) belongs to the *Flavivirus* genus of *Flaviviridae* family, and is related to other medically important flaviviruses, such as dengue (DENV), Japanese encephalitis (JEV), West Nile (WNV), and yellow fever (YFV) [1]. ZIKV was originally isolated in Uganda in 1947, and presumably expanded from Africa to Asia in the 1960s [2,3]. To date, African and Asian lineages are the two major lineages of ZIKV [4]. Before 2007, few cases of ZIKV infection were detected sporadically. The first ZIKV outbreaks occurred in Western Pacific Micronesia in 2007, and a large epidemic was recorded in French Polynesia in 2013 [5,6]. The increased pathogenicity of the Asian lineage of ZIKV might have contributed to the recent epidemics. ZIKV was introduced in Brazil in 2015, and it has rapidly spread in the Americas and Caribbean islands [7,8]. In humans, ZIKV infection was implicated in causing severe clinical consequences, including congenital malformations and neurological abnormalities [9,10]. Sexual transmission of ZIKV has been also documented [11]. The World Health Organization (WHO) declared Zika fever a serious public health emergency in 2016. 

Flaviviruses, such as ZIKV, contain a positive single-stranded RNA genome encoding a large polyprotein that is processed co- and post-translationally into three structural proteins (C, prM/M, and E) and seven non-structural proteins, NS1 to NS5 [12,13]. Glycoprotein NS1 (352 amino acids) is synthesized as a protomer, which contains six intramolecular disulfide linkages that contribute to the stabilization of the polypeptide. Alignment of different flaviviral NS1 proteins identified conserved regions [14]. As shown in Figure S1, the NS1 proteins from clinical isolate PF13/2015-18 of ZIKV and live-attenuated 17D-204 strain of YFV share at least 82% of similarity in amino acids. Once processed from the viral polyprotein into the lumen of the endoplasmic reticulum, the *N*-glycosylated NS1 protein forms a homo-dimer which acquires the capacity to associate to lipids [15]. Most of the newly synthesized NS1 dimers remain associated to viral replication complexes. However, a fraction of NS1 dimer is driven towards the plasma membrane, and another one is secreted as a hexameric lipoprotein particle. It has been recently proposed that the NS1 dimer could act as a structural intermediate that is formed during the process of NS1 hexamer formation [16]. The quaternary organization of the NS1 hexamer and the associated lipids has been recently resolved for different flaviviruses, including ZIKV [15,16,17]. The extracellular NS1 hexamer has been shown to play an important role in the pathogenesis of flavivirus-associated diseases [18,19,20]. On the other hand, the secreted NS1 is highly immunogenic during flavivirus infection, and it can also be used as a diagnostic biomarker of disease [21].

Both NS1-based vaccines and NS1-targeting antibody that inhibit NS1 activity are promising antiviral strategies to prevent flavivirus disease progression [22,23,24,25,26]. Remarkably, it has been recently demonstrated that induction of a peripheral immunity against NS1 can protect mice against a lethal challenge with ZIKV [27]. Following the observation that an NS1-based vaccine is able to mediate protection against ZIKV infection, it will therefore be a high priority to generate NS1-targeting antibodies for investigating the role of NS1 in disease development. Knowing the oligomeric nature of NS1, it is essential to dispose NS1 antibodies targeting the secreted form with aim of elucidating the role of hexameric lipoprotein particles in the ZIKV-mediated cell dysfunction. Such NS1-targeting antibodies will help to establish the NS1 function in the context of ZIKV infection, and also in the search for NS1 targeting inhibitors.

Recombinant flavivirus NS1 antigens have been used as biological tools for the immunocapture of anti-NS1 antibody [28]. Anti-NS1 monoclonal antibodies were generated, allowing the detection of soluble NS1 in the bloodstream of dengue patients during the acute phase of disease [29]. In an effort to produce specific anti-ZIKV NS1 immune serum, we reported that immunization of adult rat with a crude *Escherichia coli* extract which overexpressed the N-terminal region of recombinant NS1 (rNS1^1–151^) elicited the production of NS1 antiserum that reacts preferentially with the NS1 monomer [30]. In the present study, we used a lentiviral TRIP vector for expression of a recombinant full-length NS1 protein from ZIKV strain isolated in Brazil in 2015. Non-human primate Vero cells were stably transduced with a lentiviral vector containing the NS1 gene. We showed that immunization with the secreted recombinant NS1 dimer elicits the production of antibody against NS1 dimer.

## 2. Results and Discussion

### 2.1. Stable HEK293 and Vero Cell Lines Expressing Recombinant NS1 Protein from Zika Virus (ZIKV)

#### 2.1.1. Expression of Recombinant ZIKV NS1 Protein Using a Mammalian-Optimized Codon NS1 Gene

**Figure 1:**
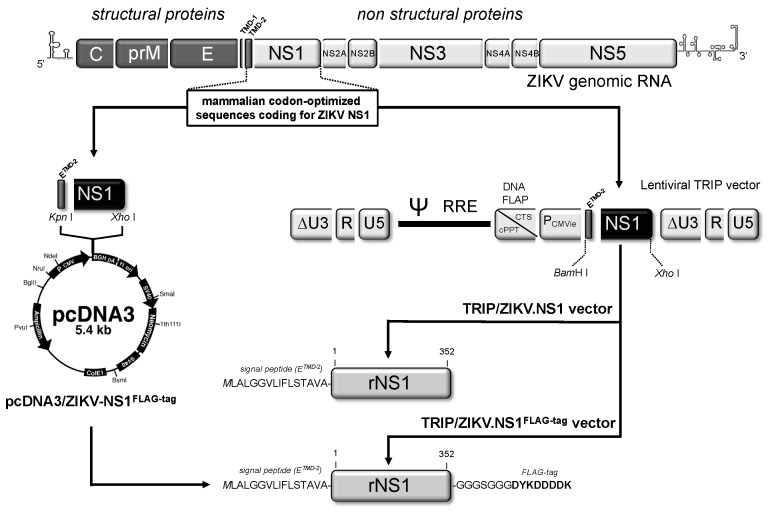
Cloning strategy of pcDNA3/ZIKV.NS1 and TRIP/ZIKV.NS1. In top, the schematic representation of Zika virus (ZIKV) genomic RNA with the E gene including the two transmembrane domains TMD-1 and TMD-2 at its C-terminus followed by the NS1 gene. In right, the mammalian codon-optimized sequence encoding TMD-2 from E followed by NS1 was cloned into the plasmid vector pcDNA3(+) under the control of human cytomegalovirus promoter (*P*_CMV_). The resulting plasmid pcDNA3/ZIKV.NS1 includes the signal peptide of NS1 (ETMD-2) and the entire NS1 gene of epidemic ZIKV strain BeH81905, followed by a glycine–serine spacer and a FLAG-tag at the C-terminus of NS1. In left, the mammalian codon-optimized sequences encoding TMD-2 from E followed by NS1 were cloned into the lentiviral TRIP vector under the control of immediate early CMV promoter (*P*_CMVie_). Recombinant TRIP/ZIKV.NS1 vector and TRIP/ZIKV.NS1^FLAG-tag^ vector include the same ZIKV sequence that pcDNA3/ZIKV.NS1 except TRIP/ZIKV.NS1 vector that has been deleted of the spacer and FLAG-tag at the C-terminus of NS1. In bottom, the design of recombinant ZIKV NS1 (rNS1) protein with its signal peptide ETMD-2 and followed or not by a spacer and a FLAG-tag is represented. The recombinant NS1 sequences are described in Figure S2.

**Figure 2:**
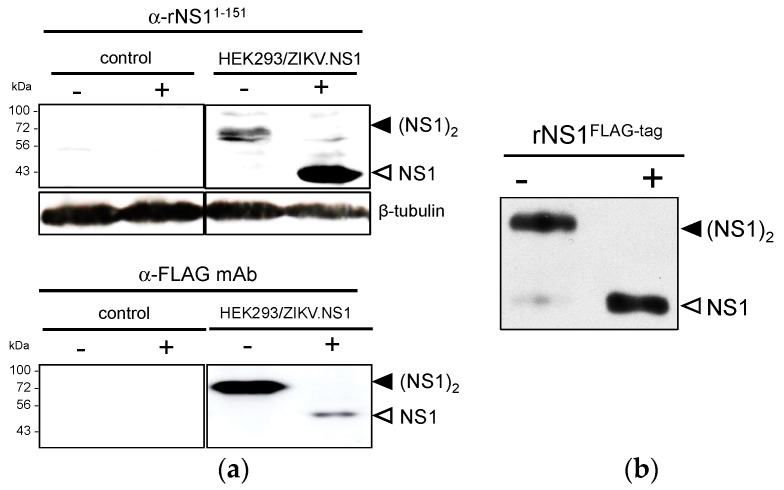
Expression of recombinant ZIKV NS1 protein in HEK-293 cells stably transfected with a plasmid vector. (**a**) Immunoblot analysis of rNS1 expression in RIPA lysates from HEK293/ZIKV.NS1^FLAG-tag^ (HEK293/ZIKV.NS1) and mock-transfected HEK293 (control) cells. Samples were heat-denatured (+) or not (−) before immunoblotting with rat anti-NS1 serum (α-rNS1^1–151^) or mouse anti-FLAG mAb (α-FLAG mAb). Detection of β-tubulin served as protein loading control; (**b**) Samples of purified rNS1^FLAG-tag^ were heat-denatured (+) or not (−) and then analyzed by immunoblotting with anti-FLAG mAb. The bands corresponding to NS1 dimer (black arrow head) and NS1 monomer (open arrow head) are indicated to the right of the blot.

In order to produce a recombinant NS1 from a contemporary epidemic strain of ZIKV, modifications that optimize the expression of a viral gene in mammalian cells were done on the original NS1 sequence of epidemic ZIKV strain BeH819015 isolated in Brazil in 2015 (Figure S2). Given that plasmid vector pcDNA3 was successfully used for the expression of DENV NS1 in human cells [31], we decided to validate the expression of the mammalian codon-optimized ZIKV NS1 gene using pcDNA3.1(+) Neo (Figure 1). The NS1 sequence was inserted into pcDNA3.1(+) Neo to generate recombinant plasmid pcDNA3/ZIKV-NS1^FLAG-tag^. In this construct, ZIKV NS1 was preceded by the second transmembrane domain of E acting as NS1 signal peptide, and ended with a FLAG-tag (Figure S2). HEK-293 cells were transfected with pcDNA3/ZIKV-NS1^FLAG-tag^, selected on growth medium supplemented with geneticin to establish a stable HEK293/ZIKV-NS1^FLAG-tag^ cell line. Immunoblot assays using anti-ZIKV rNS1^1–151^ or anti-FLAG mAb were performed on RIPA lysates of HEK293/ZIKV.NS1^FLAG-tag^ cells (Figure 2a). It has been widely reported that flavivirus NS1 glycoprotein (app. MW 48 kDa) is converted to a heat-labile dimeric form (app. MW 72 kDa) inside the infected cells [31]. Because the NS1 dimers are sensitive to heat denaturation, cell lysates were analyzed in SDS-PAGE before and after heat denaturation of the samples. The presence of heat-labile NS1 dimer was clearly detected in HEK293/ZIKV.NS1^FLAG-tag^ cells regardless the antiserum tested. Thus, transfection of HEK293 cells with pcDNA3 containing the mammalian optimized-codon of ZIKV NS1 gene leads to efficient production of rNS1^FLAG-tag^. Secreted soluble rNS1^FLAG-tag^ was purified from the extracellular medium of HEK293/ZIKV-NS1^FLAG-tag^ cells by anti-FLAG antibody-based affinity chromatography. Immunoblot assay using anti-FLAG antibody confirmed that purified ZIKV rNS1 essentially exists as a dimer (Figure 2b). These results showed that mammalian codon-optimized NS1 gene from ZIKV strain BeH819015 is suitable for the production of secreted rNS1 dimer in mammalian cells.

#### 2.1.2. Secretion of ZIKV Rns1 from Vero Cells Stably Transduced by a Lentiviral Vector

**Figure 3:**
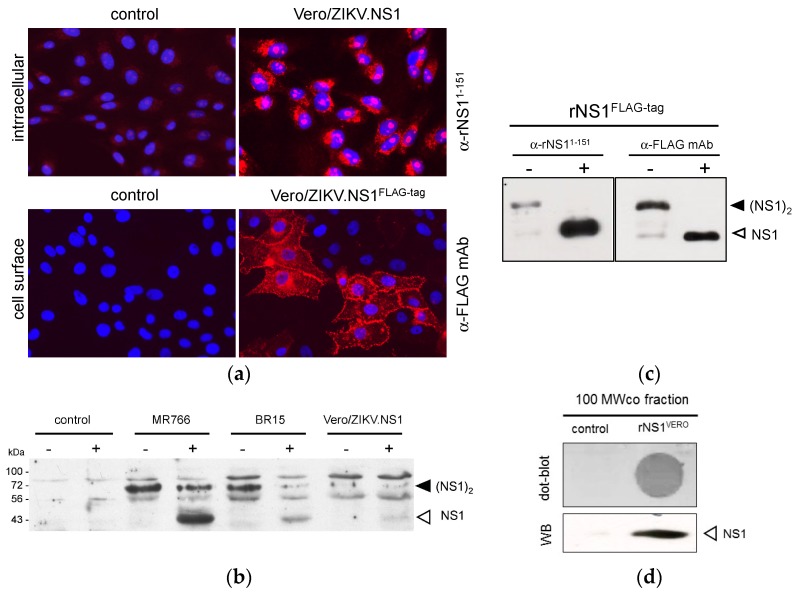
Expression of recombinant ZIKV NS1 protein in Vero cells transduced with a lentiviral vector. (**a**) IF analysis was performed on stably transduced Vero cells expressing rNS1. Fixed Vero/ZIKV.NS1 cells were permeabilized with non-ionic detergent and incubated with rat anti-NS1 immune serum (α-NS1^1-151^). Fixed Vero/ZIKV.NS1^FLAG-tag^ cells were directly stained with mouse anti-FLAG monoclonal antibody (α-FLAG mAb). Nuclei were visualized with DAPI. Mock-transduced Vero cells (control) served as a negative control; magnification: ×400 ; (**b**) Immunoblot analysis of rNS1 in RIPA lysates from Vero/ZIKV.NS1^FLAG-tag^ cells, mock-transduced Vero cells (control) and Vero cells infected 48 h with ZIKV strains MR766 or BR15. Samples were heat-denatured (+) or not (−). Authentic and recombinant NS1 were detected using rat anti-rNS1^1-151^ immune serum. Detection of β-tubulin served as protein loading control; (**c**) Samples of purified FLAG-tagged ZIKV rNS1 (NS1^FLAG-tag^) were heat-denatured (+) or not (−) and then analyzed by immunoblotting with rat anti-NS1 immune serum (α-NS1^1-151^) or mouse anti-FLAG monoclonal antibody (α-FLAG mAb) as described above. The bands corresponding to NS1 dimer (black arrow head) and NS1 monomer (open head arrow) are indicated to the right of the blot; (**d**) 100 MWco fraction samples from supernatants of Vero/ZIKV.NS1 cells (rNS1^VERO^) and Vero cells (control) were analyzed by dot-blotting (dot-blot) or Western blotting (WB) with anti-rNS1^1-150^ immune serum.

Lentiviral vectors represent an attractive platform allowing NS1 protein production in mammalian cells [32,33,34]. The mammalian codon-optimized ZIKV NS1 gene was inserted in lentiviral vector TRIP, to generate recombinant lentiviral vectors expressing NS1 with or without FLAG-tag (Figure 1). The attempts to establish stably transduced HEK293 cell lines expressing recombinant NS1 of ZIKV were unsuccessful. Consequently, we decided to transduce Vero cells to generate stable Vero/ZIKV.NS1 and Vero/ZIKV.NS1^FLAG-tag^ cell lines. By immunofluorescence analysis, both Vero/ZIKV.NS1 and Vero/ZIKV.NS1^FLAG-tag^ cells were positively stained with anti-rNS1^1–151^ immune serum and anti-FLAG mAb, respectively (Figure 3a). ZIKV rNS1 is expressed on the plasma membrane of transduced Vero cells consistent with a cell membrane interaction of NS1 dimer, as has been reported with authentic NS1 [18]. Immunoblot assays were performed on RIPA cell lysates of Vero/ZIKV.NS1 cells (Figure 3b). As positive controls, Vero cells were infected with ZIKV molecular clones derived from African strain MR766 isolated in Uganda in 1947, and Asian strain BeH819015 isolated in Brazil in 2015 (hereafter designed BR15). Anti-rNS1^1–151^ immune serum confirmed the expression of rNS1 in Vero/ZIKV.NS1 cells (Figure 3b).

We assessed whether the secretion of soluble rNS1 was efficient in Vero/ZIKV.NS1 cell lines. FLAG-tagged rNS1 protein was purified from supernatant of Vero/ZIKV.NS1^FLAG-tag^ cells by immunoaffinity chromatography, as described above. Immunoblot assay using anti-FLAG mAb showed that purified extracellular rNS1 essentially exists as a stable dimer (Figure 3c). We assessed whether the secreted rNS1 without FLAG-tag can be concentrated from the supernatants of Vero/ZIKV.NS1 cells. Extracellular medium of mock-infected Vero cells was used as a negative control. Total proteins into the supernatants of Vero/ZIKV.NS1 cell cultures were concentrated by ultrafiltration using a crossflow cassette with a cut-off of 100 kDa. As shown in Figure 3d, the remaining solution, designed hereafter as 100 MWco fraction, was positive for rNS1. Taken together, these results confirmed that transduction of Vero cells with TRIP/ZIKV.NS1 vector leads to efficient secretion of recombinant NS1, and the 100 MWco fraction is suitable for the further experiments.

### 2.2. Immunization of Rat and Anti-ZIKV NS1 Immune Serum Reactivity

#### 2.2.1. Immunization of Adult Rat with ZIKV rNS1

**Figure 4:**
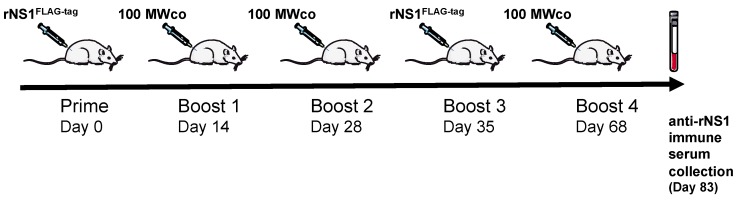
Sequence of rat immunization with recombinant ZIKV NS1 protein. Adult female Wistar rat was subcutaneously immunized with purified rNS1^FLAG-tag^. After the prime, animal was boosted with rNS1 into the 100 MWco fraction of Vero/ZIKV.NS1 cell supernatants. A second inoculation of purified rNS1^FLAG-tag^ was performed at Day 35. Whole blood was collected 83 days after the prime.

The secreted rNS1 from Vero/ZIKV.NS1 cells was used as recombinant viral antigen for immunization of adult female Wistar rat. The sequence of rat immunization is depicted in Figure 4. Induction of an antibody-based immune response against ZIKV NS1 was essentially based on a series of injections of semi-purified secreted NS1 protein (100 MWco fraction) from Vero/ZIKV.NS1 cells. In order to initiate humoral immunity towards extracellular NS1 oligomers, animals were subcutaneously primed with 5 µg of purified rNS1^FLAG-tag^. To drive the anti-NS1 humoral immune response towards the hexameric lipoprotein particles, immunized rats were boosted with 50 µg of extracellular proteins in a 100 MWco fraction. An antigenic boost with 5 µg of purified rNS1^FLAG-tag^ was performed at Day 35 to specifically enhance the production of antibody against ZIKV NS1 dimer prior the last inoculation of 100 MWco fraction at Day 68. The rat immune serum was collected at Day 83, and further analyzed for the reactivity of anti-NS1 immune serum.

#### 2.2.2. Reactivity of Anti-ZIKV rNS1 Antibodies against Authentic NS1 Protein

**Figure 5:**
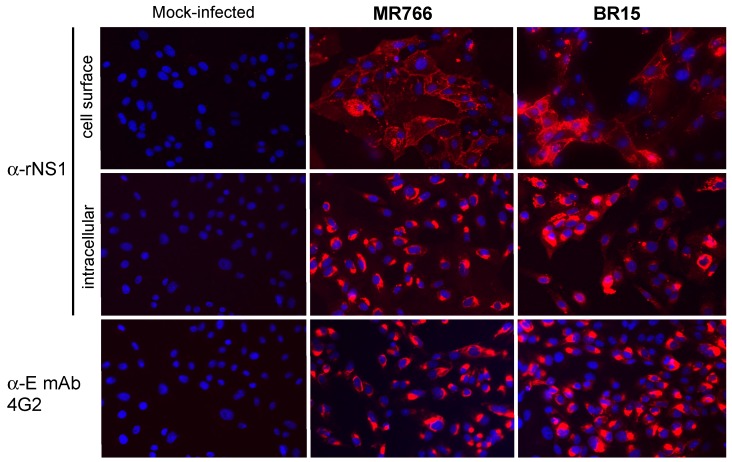
Reactivity of rat anti-rNS1 immune serum against NS1 synthesized in Vero cells infected by different strains of ZIKV. Vero cells were infected with MR766 and BR15 or mock-infected. Cells were fixed with PFA and permeabilized (intracellular) or not permeabilized (cell surface) with non-ionic detergent, and incubated with rat anti-rNS1 immune serum at dilution 1:2000. The anti-E mAb 4G2 served as a control of ZIKV infection. Nuclei were stained with DAPI. Magnification: ×400. The reactivity of rat anti-rNS1 immune serum was evaluated by immunoblotting on RIPA lysates of Vero cells infected with historical strain MR766, or epidemic strains BR15 and PF-25013-18 of ZIKV (Figure 6). The NS1 proteins from BR15 and PF-25013-18 share 100% of amino acid identity. Infection with YFV 17D strain was performed to evaluate the specificity of anti-NS1 antibody. ZIKV growth in Vero cells was confirmed by immunoblot assay using anti-E mAb 4G2 (Figure S3). Immunoblot assay using anti-rNS1^1-151^ immune serum detected both NS1 monomer and dimer in ZIKV-infected Vero cells (Figure 6a). We noted that reactivity of BR15 NS1 dimer with anti-rNS1^1–151^ immune serum was weaker when compared to historical strain MR766. Rat anti-rNS1 immune serum reacted with authentic NS1 synthesized in Vero cells infected with ZIKV but not YFV (Figure 6b). An infectious YFV titer of 4.3 log PFU.mL^−1^ was found at 24 h post-infection, confirming the productive infection of Vero cells by YF-17D.

We assessed whether the rat immune serum reacted with NS1 produced in host cells infected with ZIKV. Immunofluorescence assays were performed on Vero cells infected for 48 h with MR766 and BR15 (Figure 5). Anti-*pan* flavivirus E mAb 4G2 was used as a positive control for ZIKV infection [35]. We showed that rat immune serum collected at Day 83 strongly reacted with intracellular and plasma membrane-associated NS1, regardless the viral strains tested. Thus, hyperimmunization of rat with rNS1 elicited the production of anti-NS1 immune serum that reacts with African and Asian strains of ZIKV. 

**Figure 6:**
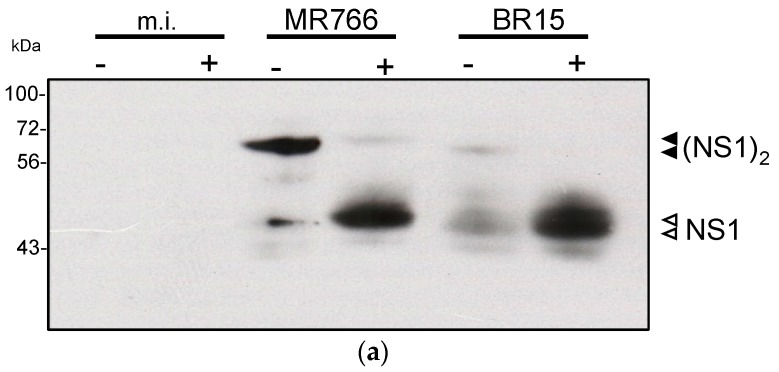

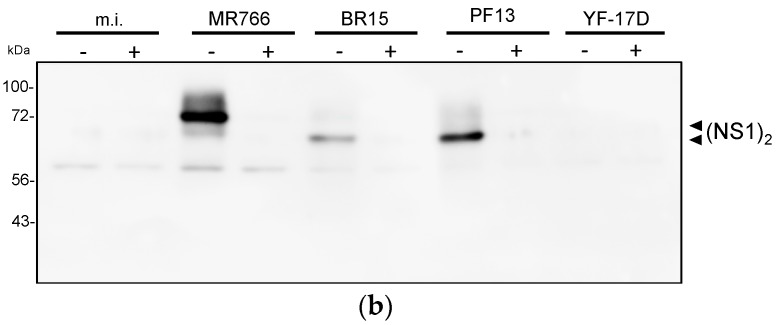
Reactivity of rat anti-rNS1 immune serum against ZIKV NS1 protein. RIPA lysates obtained from Vero cells infected with ZIKV strains MR766, BR15, and PF-25013-18 (PF13) or live-attenuated YFV 17D vaccine (YF-17D) or mock-infected (m.i.) were incubated with anti-NS1^1-151^ (**a**) or anti-rNS1; (**b**) immune serum (dilution 1:500 and 1:1000 respectively) by immunoblot assay. The samples were heat denatured (+) or not (−) before immunoblot analysis. To the right of the blot, the open head arrow indicates the position of the NS1 monomer, and the black head arrow indicates the position of the NS1 dimer. The molecular weight (MW) of protein size markers is indicated to the left of the blot.

Remarkably, only authentic NS1 dimer was detected with the rat anti-rNS1 immune serum, suggesting that majority of antibodies bind to NS1 epitopes which are exposed at the surface of NS1 oligomer (Figure 6b). We observed that both anti-rNS1^1–151^ and anti-rNS1 antisera reacted with a BR15 or PF-13-18 NS1 dimer that migrated faster that of MR766 (Figure 6). A faster migration of BR15 and PF-13-18 NS1 monomers was also observed by immunoblot assay using anti-rNS1^1–151^ immune serum (Figure 6a). There are nine amino acid substitutions at positions I21V, E52D, K69R, E146K, R191K, A194V, V236I, V264M, and Y286H between NS1 proteins from MR766 and BR15 (or PF-13-18). Whether these amino acid changes could contribute to the faster electrophoretic mobility of NS1 proteins from epidemic ZIKV strains of Asian lineage is an important issue that remains to be investigated. Altogether, these results showed that immunization with secreted rNS1 preferentially resulted in the production of immune serum essentially directed against ZIKV NS1 dimer and presumably, the NS1 hexamer. Importantly, the rat anti-rNS1 immune serum comparably recognized the NS1 dimer produced by ancestral and contemporary epidemic strains of ZIKV.

**Figure 7:**
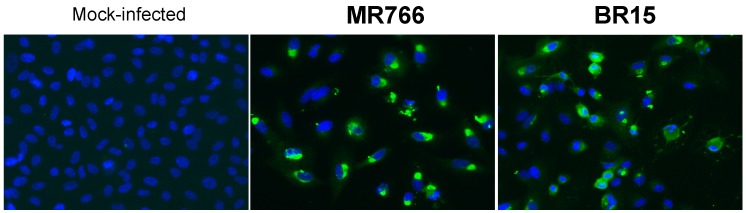
Reactivity of anti-rNS1 immune serum against ZIKV NS1 synthesized in human cells. A549 cells were infected 48 h with ZIKV strains MR766 and BR15 or mock-infected and analyzed by IF assay. Fixed cells were permeabilized with non-ionic detergent and then incubated with rat anti-rNS1 immune serum at dilution 1:2000. Magnification: ×400.

**Figure 8:**
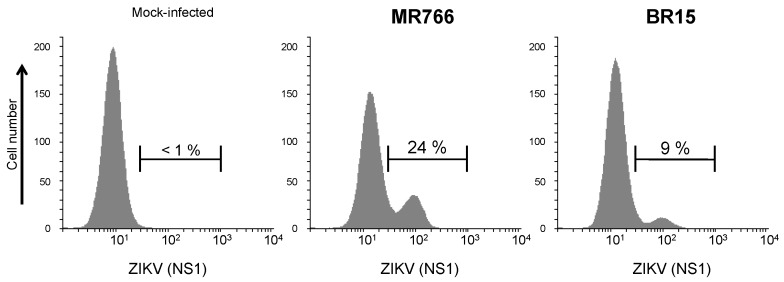
Reactivity of rat anti-rNS1 immune serum against NS1 produced in human epithelial cells infected by ZIKV. A549 cells were infected for 24 h with ZIKV strains MR766 and BR15 or mock-infected (no virus). Fixed cells were permeabilized and incubated with rat anti-rNS1 immune serum at dilution 1:1000 as primary antibody. The percentages of ZIKV-infected A549 cells positively stained with anti-rNS1 immune serum were determined by flow cytometry analysis, and the values are indicated on the top.

We assessed whether the rat anti-rNS1 immune serum recognizes intracellular NS1 produced in human lung epithelial A549 cells infected with ZIKV [28,29]. By IF analysis, rat immune serum against NS1 oligomer recognized intracellular NS1 in A549 cells infected with African historical strain MR766 or Asian epidemic strain BR15 of ZIKV (Figure 7). The immune detection of authentic NS1 protein was confirmed by flow cytometry analysis (Figure 8). These results showed that rat anti-rNS1 immune serum is suitable for the immune detection of NS1 synthesized in human cells infected with ZIKV strains of different lineages.

### 2.3. Concluding Remarks

In the last five years, mosquito-borne ZIKV has become a serious public health concern worldwide. There is an urgent need to dispose of specific and sensitive biological tools for ZIKV detection. Secreted NS1 produced in human host cells infected with ZIKV is a potential biological marker for viral infection. Authentic and recombinant NS1 proteins have been used as viral antigens for the capture of specific antibodies directed against medically important flaviviruses [21,28,29]. It has been also reported that NS1-based vaccines induce partial protective immunity against flavivirus infection [14]. Recently, we demonstrated that rat immunization with the first 151 amino acids of ZIKV NS1 expressed in bacteria resulted in the production of anti-NS1 antibodies that preferentially recognize the NS1 monomer [30]. To better understanding the role of the NS1 protein in the pathophysiology of ZIKV infection, it is necessary to dispose of specific anti-NS1 antibodies that are able to recognize both cell membrane-associated NS1 dimer and secreted NS1 hexamer. Consequently, we decided to produce a recombinant full-length NS1 protein from a contemporary epidemic strain of ZIKV using the non-replicative lentiviral TRIP vector that is particularly effective for the expression of secreted flaviviral antigens [32,33,34]. We first evaluated the ability of a mammalian-codon optimized NS1 sequence to produce an immunogenic NS1 in HEK293 cells stably transfected with a vector plasmid expressing ZIKV NS1. Immunoblot assays confirmed the expression of recombinant NS1 dimer that is released into the extracellular space, presumably as a hexamer. The ZIKV NS1 gene was therefore inserted into the lentivirus TRIP vector, and Vero cells were transduced with the recombinant lentiviral vector expressing ZIKV NS1. We showed that recombinant NS1 exists as both cell surface-associated and secreted proteins in stably transduced Vero cells. Immunization of adult rat with secreted recombinant NS1 elicited production of anti-ZIKV NS1 immune serum. Reactivity of rat anti-NS1 immune serum was documented by immunofluorescence and immunoblot assays. The NS1 antiserum specifically recognizes the NS1 dimer synthesized in host cells infected with historical African and epidemic Asian strains of ZIKV. No cross-antigenic reactivity of anti-ZIKA NS1 immune serum was observed with the NS1 synthesized by the live 17D vaccine of YFV. In conclusion, we demonstrated that recombinant ZIKV NS1 expressed in mammalian cells stably transduced with a lentiviral vector is suitable for inducing the production of specific antibody directed against NS1 oligomer. Anti-ZIKV immune serum could be a great tool for the specific detection of secreted NS1 hexamer in human cells infected with ZIKV of African and Asia lineages, including contemporary epidemic strains.

## 3. Materials and Methods

### 3.1. Cell Lines and Viruses

Monkey kidney normal Vero cells (ATCC, CCL-81), human embryonic kidney HEK-293 cells (ATCC, CRL-1573), and human carcinoma epithelial lung A549 cells (ATCC, CCL-185) were cultured at 37 °C under a 5% CO_2_ atmosphere in MEM Eagle medium supplemented with 5% to 10% of heat-inactivated fetal bovine serum (FBS, Dutscher, Brumath, France) and penicillin/streptomycin/fungizone (PAN Biotech Dutscher, Brumath, France). The clinical isolate PF-25013-18 of ZIKV has been previously described [36]. ZIKV molecular clones MR766 and BR15 were generated from African historical strain MR766-NIID (GenBank access: LC002520) isolated in Uganda in 1947 and Asian epidemic strain BeH819015 isolated in Brazil in 2015 (GenBank access: KU365778) [35]. Live-attenuated vaccine strain 17D of yellow fever virus (YF-17D STAMARIL, Sanofi-Pasteur, Lyon, France) was twice propagated in Vero cells. Virus stocks were grown on Vero cells, and their infectivity was determined by plaque-forming assays [36]. Cells were routinely infected with ZIKV strains or YF-17D virus at the multiplicity of infection of 1 plaque forming unit per cell. 

### 3.2. Stable Transfection of HEK293 Cells with Vector Plasmid Expressing NS1

The mammalian codon-optimized sequence coding for the NS1 signal peptide, followed by the amino acids 1 to 352 of NS1 protein from ZIKV strain BeH819015, was synthesized by Genecust (Luxembourg) (Figure S2). The synthetic NS1 gene was cloned into *Kpn* I and *Xho* I restriction sites of the pcDNA3.1(+) Neo plasmid (Thermo Fisher Scientific, Villebon sur Yvette, France) to generate pcDNA3/ZIKV-NS1^FLAG-tag^ (Figure 1). In this construct, a glycine–serine spacer and a FLAG-tag sequence followed the NS1 sequence. HEK293 cells were transfected with pcDNA3/ZIKV-NS1^FLAG-tag^ using Lipofectamine 3000 (Thermo Fisher Scientific). Stable HEK293/ZIKV-NS1^FLAG-tag^ cell line was selected with 800 µg·mL^−1^ of geneticin G418.

### 3.3. Stable Transduction of Vero Cells with Lentiviral Vectors Expressing NS1

The mammalian codon-optimized sequences coding ZIKV NS1 protein, with or without FLAG-tag, were cloned into the BamHI and XhoI restriction sites of the pTRIP∆U3CMV plasmid to generate pTRIP∆U3CMV/ZIKV.NS1 and pTRIP∆U3CMV/ZIKV.NS1^FLAG-tag^ (Figure 1). Lentiviral particles were produced by transient calcium co-transfection of Vero cells, as previously described [24]. The recombinant lentiviral vectors were pseudotyped with VSV-G envelope protein of serotype Indiana. In the resulting lentiviral vectors, TRIP/ZIKV.NS1 and TRIP/ZIKV.NS1^FLAG-tag^, the early promoter immediately (P_CMVie_) drive the constitutive expression of recombinant NS1 of ZIKV (Figure 1). Stable Vero/ZIKV.NS1 and Vero/ZIKV.NS1^FLAG-tag^ cell cultures were obtained by transduction of Vero cells with TRIP/ZIKV.NS1 or TRIP/ZIKV.NS1^FLAG-tag^, respectively.

### 3.4. Production and Purification of Recombinant NS1

To semi-purify secreted rNS1, Vero/ZIKV.NS1, cell monolayers were cultured for 4 days with synthetic Panserin 401 medium (PAN Biotech) that has been developed for serum-free cell cultivation. Non-denaturing concentration of rNS1 dimer in cell supernatant was realized using a two-step concentration and diafiltration method. The culture medium (500 mL) was passed through a Vivaflow 200 system with a cut-off of 100 kDa (Sartorius) and the remaining solution was then reduced to a final volume of 10 mL on a Vivacell cassette with a cut-off of 50 kDa (Sartorius). The 100 MWco fraction contained a 50-fold concentration of the constituent proteins with a molecular weight greater than 100 kDa from the original culture medium. The samples were verified for the presence of rNS1 by immunoblot assay, and stored at −80°C after a flash frozen in liquid nitrogen. Protein quantification in the 100 MWco fraction was performed using BCA assay (Sigma-Aldrich).

To purify secreted FLAG-tagged rNS1, supernatants of HEK293/ZIKV.NS1^FLAG-tag^ and Vero/ZIKV.NS1^FLAG-tag^ cell monolayers cultured 4 days with synthetic Panserin 401 medium were clarified by centrifugation for 10 min at 1200*g* and subjected to diafiltration as above. The remaining solutions contained a 10-fold concentration of the constituent proteins from the original culture medium. The solutions were loaded on an anti-FLAG M2 affinity agarose gel column (Sigma-Aldrich, Saint-Quentin-Fallavier, France). Bound FLAG-tagged proteins were eluted from affinity column with 100 µg·mL^−1^ FLAG peptide in PBS (Sigma-Aldrich). The elution samples were dialyzed against PBS to remove the unbound FLAG peptide, and analyzed using 8% SDS-PAGE and further processed using PlusOne Silver staining kit (GE Healthcare, Buc, France). The samples containing the purified rNS1^FLAG-tag^ were quantified and stored as above.

### 3.5. Rat Experiments and Ethical Statement

Experiments were conducted following the guidelines of Office Laboratory of Animal Care at the CYROI platform. Rats were housed individually in separate cages at the CYROI platforms’ animal facility. The CETEA at the CYROI has ethically validated the protocols and subsequent experiments in the document n°A974001, which has been then approved by the French authorities (03197.02, 18 July 2016).

Adult Wistar rats were serially subcutaneously inoculated with rNS1 in PBS mixed with Complete Freund’s Adjuvant (prime immunization) or Incomplete Freund’s Adjuvant (the antigenic boosts) in a final volume of 0.2 mL. Three months after the prime, whole blood was collected by cardiac puncture after rats were anesthetized. 

### 3.6. Immunoblotting

For Western blot assays, samples were processed by 10% SDS-PAGE, and transferred onto a nitrocellulose membrane. After blocking of the membrane for 1 h with 90% FBS in PBS-Tween, the blot was incubated overnight at 4 °C with appropriate dilution of the primary antibody in the same buffer. Rat immune sera directed against rNS1^1–151^ and rNS1 were used at dilutions 1:500 and 1:1000, respectively. Mouse mAb anti-FLAG tag (Sigma Aldrich) was used at dilution 1:1000. Anti-mouse and anti-rat IgG HRP-conjugated secondary antibodies were used at 1:2000 dilution (Vectors Labs, Premanon, France). The membranes were developed with Pierce ECL Western blotting substrate (Thermo Fisher Scientific) and exposed on an Amersham imager 600 (GE Healthcare). For dot blot assays, the sample was directly loaded on a nitrocellulose membrane and then probed with antibodies as described above.

### 3.7. Immunofluorescence and Flow Cytometry Assays

For IF analysis, cell monolayers grown on glass coverslips were incubated 20 min with paraformaldehyde (PFA) at the final concentration of 3.2% in PBS. When required, permeabilization of fixed cells was performed for 4 min with nonionic detergent Triton X-100 at the final concentration of 0.1% in PBS. Cells were incubated for 30 min with primary antibody at the appropriate dilution in PBS containing 1% bovine serum albumin (PBS-BSA). Rat immune sera directed against rNS1^1-151^ and rNS1 were used at dilutions 1:200 and 1:500, respectively. Mouse mAb anti-FLAG-tag was used at dilution 1:500. Mouse anti-*pan* flavivirus E protein 4G2 mAb was used at dilution 1:1000. Goat anti-mouse Alexa Fluor 594 IgG, donkey anti-rat Alexa Fluor 594 IgG and goat anti-rat Alexa Fluor 488 IgG were used as secondary antibodies at the dilution (1:2000). Nuclei were stained with DAPI. Vectashield reagent (Vector Labs, Premanon, France) was used for the mounting of the glass coverslips. A Nikon Eclipse E2000-U microscope was used for the visualization of the fluorescence. The capture of the fluorescent signal was allowed with a Hamamatsu ORCA-ER camera coupled to the imaging software NIS-Element AR (Nikon, Champigny-sur-Marne, France).

For flow cytometry analysis, cells were fixed with 3.7% PFA in PBS, permeabilized with ethanol and then blocked with PBS-BSA. Cells were incubated 20 min with rat anti-rNS1 immune serum diluted 1:1000 in PBS-BSA as primary antibody. Goat anti-rat Alexa Fluor 488 IgG at dilution 1:1000 was used as secondary antibody. Expression of ZIKV NS1 protein was analyzed by flow cytometric analysis using FACScan flow cytometer (Becton Dickinson, Le Pont-de-Claix, France).

## Supplementary Materials

Supplementary materials can be found at www.mdpi.com/1422-0067/19/1/38/s1.

## Abbreviations

HEK293 cells	human embryonic kidney 293 cells
IF	immunofluorescence
kDa	kilodalton
mAb	monoclonal antibody
MW	molecular weight
NS1	non structural protein 1
RIPA	radioimmunoprecipitation assay
SDS-PAGE	sodium dodecyl sulfate-polyacrylamide gel electrophoresis
ZIKV	zika virus
