# Supplementary material for: Recombinant Zika NS1 Protein Secreted from Vero Cells Is Efficient for Inducing Production of Immune Serum Directed against NS1 Dimer
Source: Int J Mol Sci. 2017 Dec 23;19(1):38. doi: 10.3390/ijms19010038 (PMC5795988; doi:10.3390/ijms19010038)
Supplement: Supplementary file 1 [file ijms-19-00038-s001.pdf]

Supplementary Materials

```
ZIKV     DVGCSVDFSKKETRCGTGVFVYNDVEAWRDRYKYHPDSPRRLAAAVKQAWEDGICGISSV  60
         | ||  :|:|:| :||:| |  :|:::| ::|:|:| :|::||  ||:  | |:||::||
YFV      DQGCAINFGKRELKCGDGIFIFRDSDDWLNKYSYYPEDPVKLASIVKASFEEGKCGLNSV  60

ZIKV     SRMENIMWRSVEGELNAILEENGVQLTVVVGSVKNPMWRGPQRLPVPVNELPHGWKAWGK  120
         :: |::||||:::|:|||:||| |:: ||| : ||:: ||:: :::::::|::||| |||
YFV      DSLEHEMWRSRADEINAIFEENEVDISVVVQDPKNVYQRGTHPFSRIRDGLQYGWKTWGK  120

ZIKV     SYFVRAAKTNNSFVVDGDTLKECPLKHRAWNSFLVEDHGFGVFHTSVWLKVREDYSLECD  180
         : : :  ::|:||  ||: :||||:::|:||||: |  |:|||:|:|:::::: | ::||
YFV      NLVFSPGRKNGSFIIDGKSRKECPFSNRVWNSFQIEEFGTGVFTTRVYMDAVFEYTIDCD  180

ZIKV     PAVIGTAVKGKEAVHSDLGYWIESEKNDTWRLKRAHLIEMKTCEWPKSHTLWTDGIEESD  240
         :  :| ||:||: :|:::  |::|: :: ::: :  :::::: : :  :::  :::|||
YFV      GSILGAAVNGKKSAHGSPTFWMGSHEVNGTWMIHTLEALDYKECEWPLTHTIGTSVEESE  240

ZIKV     LIIPKSLAGPLSHHNTREGYRTQMKGPWHSEELEIRFEECPGTKVHVEETCGTRGPSLRS  300
         :::|:|: ||:|:||: :||: | :|||: ::||:::|:||||:|  :::|: ||:|:||
YFV      MFMPRSIGGPVSSHNHIPGYKVQTNGPWMQVPLEVKREACPGTSVIIDGNCDGRGKSTRS  300

ZIKV     TTASGRVIEEWCCRECTMPPLSFRAKDGCWYGMEIRPRKEPESNLVRSMVTA  352
         ||:||:||:|||||:|||||:|||: :||||| |||||||::||:||||:|||
YFV      TTDSGKVIPEWCCRSCTMPPVSFHGSDGCWYPMEIRPRKTHESHLVRSWVTA  352
```

Figure S1. Comparative analysis of ZIKV and YFV NS1 proteins. Sequence alignment of NS1 (352 amino acids) from clinical isolate PF13/25013-18 of ZIKV (Genbank access number KX369547) and live attenuated 17D-204 strain of YFV (Genbank access number KF693015). The conserved sequences are shown by bar (I) and amino acids that are strongly similar are indicated by a double dot (:). The analysis was performed unsing SerialCloner software.

atgCTCGCTCTCGGAGGAGTGCTCATCTTCCTGTCCACCGCTGTGTCCGCTGATGTGGGATGTTCCGTGGATTTCTCCAA
GAAGGAGACCCGCTGCGGAACCGGAGTGTTCGTCTACAACGACGTGGAGGCATGGAGAGACAGGTACAAGTACCACCCTG
ACTCCCCCAGGCGCTTGGCTGCAGCAGTCAAGCAGGCCTGGGAAGATGGAATCTGTGGGATCTCCTCCGTGTCACGGATG
GAGAACATCATGTGGCGGTCCGTGGAGGGGGAATTGAACGCCATCCTTGAGGAGAACGGGGTGCAGCTTACCGTCGTGGT
GGGGTCTGTCAAAAACCCTATGTGGCGGGGCCCACAAAGACTCCCTGTGCCTGTGAACGAACTTCCTCACGGCTGGAAGG
CCTGGGGGAAATCTTACTTCGTCCGGGCCGCCAAGACAAACAACTCCTTCGTCGTGGATGGCGATACCTTGAAGGAGTGC
CCACTGAAGCACCGGGCCTGGAATTCTTTTCTGGTGGAGGATCACGGGTTTGGGGTCTTTCACACCAGCGTCTGGCTCAA
GGTGCGGGAGGACTACAGCCTGGAGTGCGATCCAGCCGTGATCGGGACAGCCGTGAAAGGGAAGGAAGCCGTCCATAGTG
ATCTGGGCTACTGGATCGAGAGCGAGAAGAACGACACCTGGAGGTTGAAGAGGGCCCACCTGATCGAGATGAAGACCTGC
GAGTGGCCAAAGAGCCACACACTGTGGACAGACGGGATTGAGGAGAGCGACCTGATTATTCCCAAGAGCCTGGCCGGGCC
ACTCAGCCACCACAACACACGGGAGGGCTACAGAACACAGATGAAGGGGCCCTGGCATAGCGAGGAGCTGGAAATTAGGT
TTGAGGAGTGCCCCGGCACTAAGGTCCATGTGGAGGAGACATGCGGCACTCGGGGCCCCAGTCTGAGAAGTACAACTGCC
TCAGGCAGAGTGATTGAGGAGTGGTGCTGCAGGGAGTGCACAATGCCCCCCCTGTCATTTAGGGCCAAGGACGGCTGTTG
GTATGGCATGGAGATTCGGCCCAGGAAAGAACCCGAAAGCAACCTGGTCAGGAGCATGGTCACTGCCGGCAGCACTGGCG
GCGGCAGCGGCGGCGGCGACTACAAAGACGATGACGACAAGTAATGACTCGAGtaatga

M*LALGGVLIFLSTAVSA***DVGCSVDFSKKETRCGTGVFVYNDVEAWRDRYKYHPDSPRRLAAAVKQAW
EDGICGISSVSRMENIMWRSVEGELNAILEENGVQLTVVVGSVKNPMWRGPQRLPVPVNELPHGWKA
WGKSYFVRAAKTNNSFVVDGDTLKECPLKHRAWNSFLVEDHGFGVFHTSVWLKVREDYSLECDPAV
IGTAVKGKEAVHSDLGYWIESEKNDTWRLKRAHLIEMKTCEWPKSHTLWTDGIEESDLIIPKSLAGPLS
HHNTREGYRTQMKGPWHSEELEIRFEECPGTKVHVEETCGTRGPSLRSTTASGRVIEEWCCRECTMP
PLSFRAKDGCWYGMEIRPRKEPESNLVRSMVTAGST**GGGSGGGDYKDDDDK

Figure S2. Sequences of ZIKV NS1 genes and translated proteins used in this study. In top, sequence of mammalian-codon optimized NS1 gene derived from ZIKV strain BeH819015 (Genebank access : KU365778). The sequence encoding the glycine–serine spacer followed by a FLAG tag is underlined. The exogenous initiation and terminaison codons of NS1 sequence are indicated in small letters. In bottom, the sequence of recombinant full-length NS1 protein is indicated in bold. The signal peptide of NS1 is indicated in italic. The sequence of the glycine–serine spacer followed by a FLAG tag at the C-terminus of NS1 is underlined.

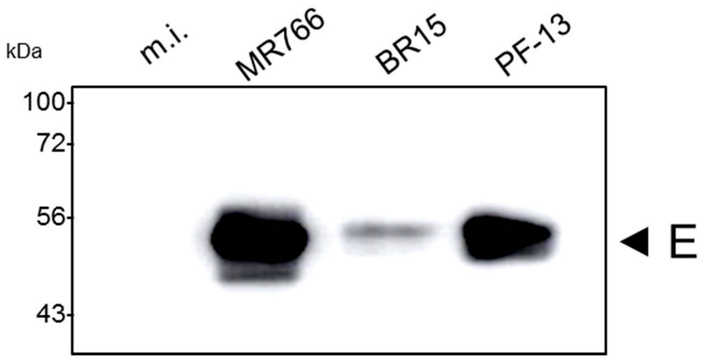

Figure S3. Detection of intracellular E protein in ZIKV-infected Vero cells. The lysates of ZIKV-infected Vero cells or mock-infected cells that are described in Fig.6 were probed with anti-E Mab 4G2.
